# Modeling the Distribution of New MRI Cortical Lesions in Multiple Sclerosis Longitudinal Studies

**DOI:** 10.1371/journal.pone.0026712

**Published:** 2011-10-20

**Authors:** Maria Pia Sormani, Massimiliano Calabrese, Alessio Signori, Antonio Giorgio, Paolo Gallo, Nicola De Stefano

**Affiliations:** 1 Biostatistics Unit, Department of Health Sciences, University of Genoa, Genoa, Italy; 2 Department of Neurosciences, The Multiple Sclerosis Center, University Hospital of Padua, Padua, Italy; 3 Department of Neurological and Behavioral Sciences, University of Siena, Siena, Italy; Washington University, United States of America

## Abstract

**Objective:**

Recent studies have shown the relevance of the cerebral grey matter involvement in multiple sclerosis (MS). The number of new cortical lesions (CLs), detected by specific MRI sequences, has the potential to become a new research outcome in longitudinal MS studies. Aim of this study is to define the statistical model better describing the distribution of new CLs developed over 12 and 24 months in patients with relapsing-remitting (RR) MS.

**Methods:**

Four different models were tested (the Poisson, the Negative Binomial, the zero-inflated Poisson and the zero-inflated Negative Binomial) on a group of 191 RRMS patients untreated or treated with 3 different disease modifying therapies. Sample size for clinical trials based on this new outcome measure were estimated by a bootstrap resampling technique.

**Results:**

The zero-inflated Poisson model gave the best fit, according to the Akaike criterion to the observed distribution of new CLs developed over 12 and 24 months both in each treatment group and in the whole RRMS patients group adjusting for treatment effect.

**Conclusions:**

The sample size calculations based on the zero-inflated Poisson model indicate that randomized clinical trials using this new MRI marker as an outcome are feasible.

## Introduction

In patients with multiple sclerosis (MS), the number of brain white matter (WM) lesions as detected by magnetic resonance imaging (MRI) is widely used as a marker for assessing and monitoring disease activity. The negative binomial (NB) distribution is known as the statistical model best fitting the number of WM lesions [1], [2]. Recent pathological studies have shown that lesions are often located in the grey matter of MS brains, especially in the cerebral cortex [3], [4]. Cortical lesions (CLs) have been detected *in vivo* by means of specific MR sequences in many research studies [5], [6]. These have clearly shown the clinical relevance of CLs, suggesting that they could become soon a valid outcome in MS studies, adding to MRI WM lesions in assessing disease activity and response to therapy [5], [6], [7].

The increased clinical relevance of CLs makes it important to know the statistical properties of the distribution of CLs across a population of MS patients for future trial design. These might be different from those of WM lesions and need to be assessed separately. Recently, the cross-sectional distribution of CLs was studied in a group of 44 relapsing remitting (RR) MS patients and the best model fitting their distribution across subjects was the NB model [8]. However, for the appropriate design of future longitudinal studies and clinical trials, it would be relevant to know the distribution of new CLs longitudinally developed by MS patients over the follow up period. Thus, in this study we analysed the best statistical model fitting the distribution of new MRI CLs developed over 1 and 2 years by a group of RRMS patients who were either untreated or treated with 3 different disease modifying drugs. Using this dataset, we also estimated the sample size for trials using MRI-derived CLs as the primary outcome.

## Methods

### Patients

The dataset comprised 191 RRMS patients, 50 who remained all the 2 years of the follow up period with no treatment and 141 that were part of a clinical study, randomized to subcutaneous (s.c.) interferon (IFN) beta-1a (44 mcg three times weekly, 46 patients), intramuscular (i.m.) IFN beta-1a (30 mcg weekly, 47 patients) or glatiramer acetate (GA; 20 mg daily, 48 patients). All patients were evaluated by MRI at baseline, 12 and 24 months. Additional trial details including design, inclusion/exclusion criteria, patients' clinical and MRI characteristics are reported extensively elsewhere [9].

The study received approval from the Ethics Committee of the University Hospital of Padua and informed written consent was obtained from all subjects.

### MRI

Images were acquired using a 1.5 T scanner (Achieva, Philips Medical Systems, Best, The Netherlands) with 33 mT/m power gradient and a 16-channel head coil. Details about MRI acquisition procedures are reported previously [9]. Briefly, double inversion recovery (DIR) acquisition parameters were the following: repetition time (TR) = 15631 ms; echo time (TE) = 25 ms; inversion time (TI) = 3400 ms; delay¼ = 325 ms; echo train length (ETL) = 17; 50 contiguous axial slices with thickness = 3 mm; matrix size¼ = 130×256; and field of view (FOV) = 250×200 mm^2^.

Imaging was carried out at the imaging centre of the University of Padua, Italy, and all images were assessed by the consensus of two experienced observers (MC and PG) who were blinded to the patients' identity and treatment. The number of new CLs was counted on the 12 and 24 month scans as compared to the baseline scan.

### Statistical analysis

Four models were fitted to the distribution of the number of new CLs counted over 12 and 24 months: the basic Poisson and the basic NB model, the zero-inflated Poisson (ZIP) and the zero-inflated NB (ZINB). The Poisson and the NB models are well known and were extensively described previously [1], [2]. Zero-inflated distributions are interesting models that have the capability of distinguishing between the so-called “structural zeros” (i.e., zero counts that are somehow inevitable), and sampling zeros, (i.e., zero counts occurring by chance) and thus they are two-component mixture models combining a proper probability distribution for counts with a portion of extra-counts located on zero. Testing zero-inflated models was motivated by the fact that CLs are expected to be less numerous than WM lesions, so more zeros are expected in their distribution. The 4 models were evaluated separately and on the whole dataset adjusting for treatment arm. Goodness of fit of different models was evaluated by the log-likelihood and the Akaike Information Criterion (AIC) and compared by the likelihood ratio (LR) test for nested models and the Vuong test for non-nested models. Model parameters were estimated using R (http://www.R-project.org).

Sample size for active controlled trials using the number of new CLs over 1 and 2 years as the primary outcome was estimated. The control arm was assumed to be made up of patients treated with s.c. IFN beta-1a or i.m. IFN beta-1a or GA. Sample size was estimated resampling from the distribution that gave the best fit to count data in each treatment group and assuming different benefit (ranging between a 30% and 50% lesion reduction) of the new drug compared to the active control (power = 90% and significance level = 5%).

## Results

**Figure 1:**
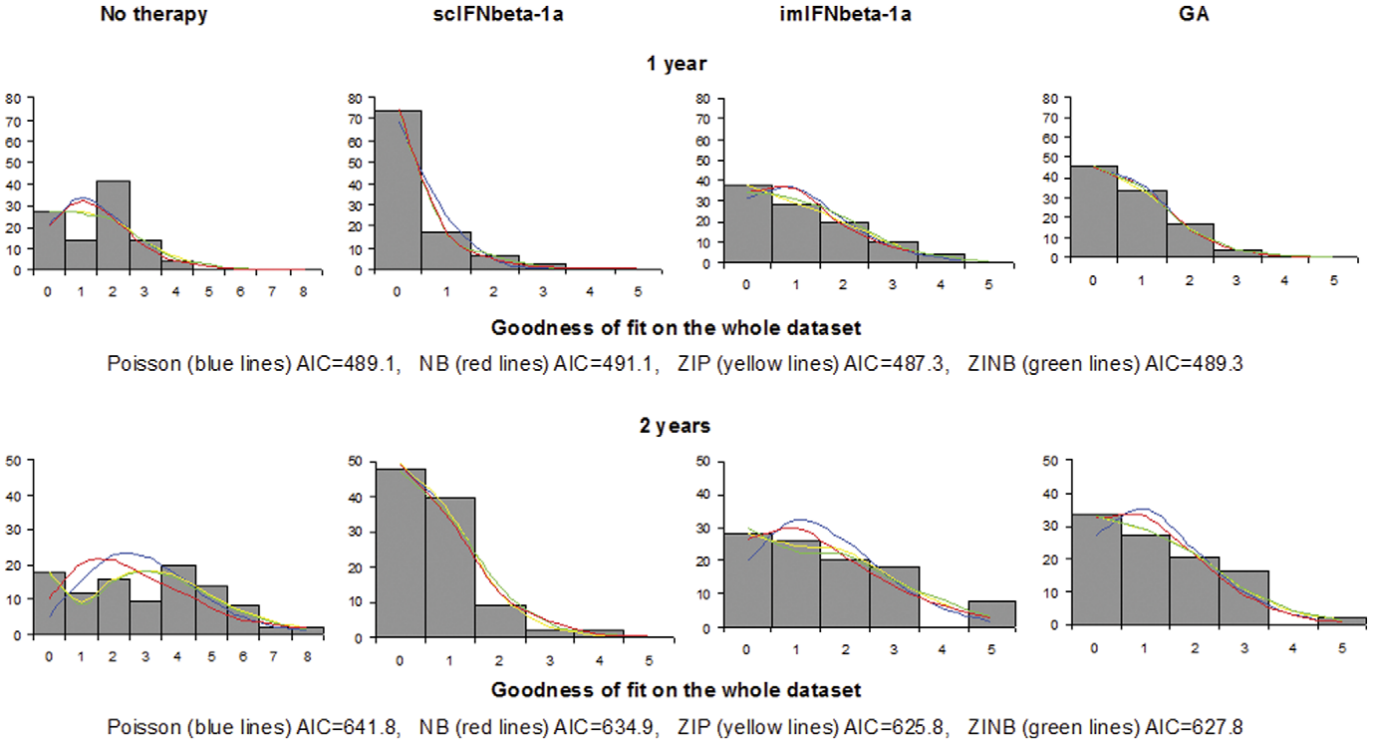
Histograms of the observed and predicted distribution of CLs over 1 and 2 years. Histograms of the distribution of the number of cortical lesions counted over 1 and 2 years in the non-treated and in the 3 treated groups, and their probability distribution implied by the Poisson (blue lines), the zero-inflated Poisson (yellow lines), the Negative Binomial (red lines) and the zero-inflated Negative Binomial (green lines) models. The Akaike Information Criterion (AIC) is calculated on the whole group of patients adjusting for treatment arm. Lower values of the AIC indicate better fits.

The descriptive statistics of the number of new CL counts over 1 and 2 years in the 3 treatment arms is reported elsewhere [9]. The distributions of new CL counts over 1 and 2 years for each treatment group are reported in Figure 1 (grey bars). The 4 models fitting is equivalent in some cases, with a better fit for the zero-inflated models (yellow line = ZIP, green line = ZINB) as compared to the simple Poisson and NB models (blue and red lines respectively), especially for 2-year data. Fitting the 4 distributions to the whole set of data adjusting for treatment arm indicates that the ZIP model (AIC = 487.3 over 1 and AIC = 625.8 over 2 years) gives the best fit to new CLs distribution. The AIC for 1 and 2 years were 489.1 and 641.8 for the Poisson model, 491.1 and 625.5 for the NB model and 489.3 and 627.8 for the ZINB model. The formal models evaluation and the parameters of the regression analysis are reported in Table 1 and 2. In Table 1 the frequencies of new MRI CLs over 1 and 2 years observed and predicted under the Poisson, the NB, the ZIP and the ZINB models are reported. A regression model based on these 4 distributions and adjusting for treatment arm was applied to the full dataset. The beta coefficients and their standard errors (SE) estimated by the regression models are reported in Table 2. For the zero-inflated models the extra-zeros parameter was not significantly different between treatment arms (both for 1 and for 2 years of follow up) and therefore a common parameter was estimated (% of extra-zeros in Table 2). The AIC criterion indicates the best fit adjusting for the number of parameters used by each model. Both for 1 and 2 years data, the best fit is given by the ZIP model.

**Table 1:** Frequencies of new MRI CLs over 1 and 2 years observed and predicted under the Poisson, the NB, the ZIP and the ZINB models.

		Frequency (%)				
1 year	Number of CLs	Observed	Poisson	ZIP	NB	ZINB
**No therapy**	0	27.5	21.7	27.6	21.7	27.6
	1	13.7	33.1	27.0	33.1	27.0
	2	41.2	25.3	23.3	25.3	23.3
	3	13.7	12.9	13.5	12.9	13.5
	4	3.9	4.9	5.8	4.9	5.8
**s.c. IFN beta-1a**	0	73.9	69.1	73.7	74.5	73.7
	1	17.4	25.6	17.7	18.3	17.7
	2	6.5	4.7	6.6	4.7	6.6
	3	2.2	0.6	1.7	1.5	1.7
**i.m. IFN beta-1a**	0	38.0	32.0	37.9	34.4	34.0
	1	28.0	36.5	28.8	35.7	30.9
	2	20.0	20.8	20.0	18.3	22.2
	3	10.0	7.9	9.2	7.7	8.7
	4	4.0	2.3	3.1	3.0	3.1
**GA**						
	0	45.8	45.3	46.1	45.3	46.1
	1	33.3	36.2	33.8	35.3	35.0
	2	16.7	14.2	15.0	14.2	14.2
	3	4.2	3.8	3.8	3.8	3.8

		Frequency (%)				
2 years	Number of CLs	Observed	Poisson	ZIP	NB	ZINB
**No therapy**	0	17.6	5.2	21.3	10.1	21.3
	1	11.8	15.3	12.6	20.4	12.6
	2	15.7	22.7	18.6	21	18.6
	3	9.8	22.4	18.4	16.4	18.4
	4	19.6	16.6	13.6	12.9	13.6
	5	13.7	9.8	8.0	7.6	8.0
	6	7.8	4.8	4.0	4.3	4.0
	7	2.0	2.0	1.7	2.5	1.7
	8	2.0	0.8	0.6	2	0.6
**s.c. IFN beta-1a**	0	47.8	48.7	49.2	48.6	46.7
	1	39.1	35.0	35.0	33.1	34.0
	2	8.7	12.6	12.6	12.6	14.4
	3	2.2	3.0	3.0	4.6	3.8
	4	2.2	0.5	0.5	1.0	1.0
**i.m. IFN beta-1a**	0	28.0	20.2	28.0	26.4	29.5
	1	26.0	32.3	24.4	29.8	22.8
	2	20.0	25.8	22.9	21.2	21.8
	3	18.0	13.8	14.3	11.9	13.7
	4	0.0	5.5	6.7	6.2	7.9
	5	8.0	1.8	2.5	2.7	2.9
**GA**	0	33.3	27.3	33.1	32.2	33.1
	1	27.1	35.5	28.7	33.0	28.7
	2	20.8	23.0	21.6	21.0	21.6
	3	16.7	9.9	10.9	9.2	10.9
	4	0.0	3.2	4.1	3.0	4.1
	5	2.1	0.8	1.2	1.1	1.2

**Table 2:** Coefficients of the regression models on the whole dataset.

		Poisson	Negative Binomial	Zero-inflated Poisson	Zero-inflated Negative Binomial
**1 year**	**Intercept**		0.43 (0.11)	0.56 (0.13)	0.56 (0.13)
	**Treatment arm**				
	No therapy	ref	ref	ref	Ref
	s.c. IFN beta-1a	−1.42 (0.27)	−1.42 (0.27)	−1.41 (0.28)	−1.41 (0.28)
	i.m. IFN beta-1a	−0.25 (0.18)	−0.25 (0.18)	−0.24 (0.19)	−0.24 (0.19)
	GA	−0.66 (0.20)	−0.66 (0.20)	−0.66 (0.21)	−0.66 (0.21)
	Overdispersion (1/ϑ)	−	0.00005	−	0.00004
	% of extra-zeros	−	−	13%	13%
	**Log-likelihood**	−240.5	−240.5	−238.7	−238.7
	**AIC**	489.1	491.1	487.3	489.3
	Poisson vs Negative Binomial, p = 0.98				
	Poisson vs Zero-inflated Poisson, p = 0.15				
	Negative Binomial vs Zero-inflated Poisson = 0.14				
	Negative Binomial vs Zero-inflated Negative Binomial, p = 0.15				
**2 years**	**Intercept**	1.09 (0.08)	1.09 (0.10)	1.25 (0.09)	1.25 (0.09)
	**Treatment arm**				
	No therapy	ref	ref	ref	ref
	s.c. IFN beta-1a	−1.42 (0.19)	−1.42 (0.21)	−1.44 (0.20)	−1.44 (0.20)
	i.m. IFN beta-1a	−0.58 (0.14)	−0.58 (0.17)	−0.58 (0.15)	−0.58 (0.15)
	GA	−0.83 (0.15)	−0.83 (0.18)	−0.84 (0.16)	−0.84 (0.16)
	Overdispersion (1/ϑ)	-	0.21	-	0.00006
	% of extra-zeros	-	-	15%	15%
	**Log-likelihood**	−316.9	−312.4	−307.9	−307.9
	**AIC**	641.8	634.9	625.8	627.8
	Poisson vs Negative Binomial, p = 0.002				
	Poisson vs Zero-inflated Poisson, p = 0.025				
	Negative Binomial vs Zero-inflated Poisson = 0.05				
	Negative Binomial vs Zero-inflated Negative Binomial, p = 0.05				

Sample sizes for active controlled trials using the number of new CLs detected over 1 and 2 years were therefore estimated assuming a ZIP distribution for lesion counts (Table 3). Sample sizes, calculated for an active controlled trial with s.c. IFN beta-1a, i.m. IFN beta-1a or GA as comparator, a power of 90% and a significance level of 5%, assuming a treatment effect of 50% ranged from 72 to 200 patients per arm for a 1 year trial and from 48 to 110 for a 2-year trial. If the minimum detectable treatment effect is assumed to be 30%, the sample size needed ranged from 212 to 630 patients per arm for a 1 year trial and from 150 to 320 for a 2-year trial.

**Table 3:** Number of patients per arm for an active controlled trial with the number of new cortical lesions counted over 1 and 2 years.

		Active control arm		
Treatment effect		i.m. IFN	GA	s.c. IFN
1 year	30%	212	300	630
	40%	110	150	360
	50%	72	100	200
2 years	30%	150	182	320
	40%	80	100	170
	50%	48	54	110

## Discussion

The number of new MRI CLs detected on yearly scans of RRMS patients under different treatment conditions is distributed according to a skewed distribution typical of counts. The ZIP model gave the best fit to the data.

The ZIP model is made by a mixture of the simple Poisson model, whose parameter has a very straightforward interpretation (mean value of lesions), and a proportion of “extra-zeros” that account for overdispersion. These extra-zeros, namely excess of subjects with zero new CLs, can be interpreted as a quote of patients with “structural” zeros, that is, subjects who have zero CLs not by chance, but because, for some reason, they do not develop this kind of lesions. Thus, when used in the presence of a treatment reducing the number of new CLs, the ZIP model is able to discriminate if the new treatment has an effect in reducing the mean value of lesions or in increasing the proportion of patients with zero lesions, or both. This is an interesting issue to address that would allow to better understanding the mechanism of action of a new drug.

Since the ZIP model depends on 2 parameters (the mean value and the extra-zeros), this is more convenient than the ZINB model, which gave in the present study a data fitting as good as the ZIP model, but after an estimation of 3 parameters (mean value, overdispersion and extra-zeros).

It is worth to stress here some limitations of the study. First, in the present analysis CLs were detected on images acquired at 1.5 T, which might not be the best approach to assess CLs (i.e. higher fields might be better to evaluate this type of lesions). However, MR scanners at 1.5 T field strength are still the most widely used in clinics and are those that will most likely be used for large clinical trials in the near future. Second, new CLs are not yet considered as a relevant surrogate marker of disease in MS. However, since the development of new agents acting on neuroprotection and repair is a hot topic in the MS research, there is the consequent need of new MRI outcomes for testing these drugs in phase II studies. Among others, CLs are a promising MRI outcome for neuroprotection and repair [10] and the present analysis gives the technical basis for using them in future phase II studies. Finally, since CLs are less frequent than WM lesions, the sample size needed for a clinical trial is presumed to be very large, making the trial unfeasible. In this study, by fitting the optimal ZIP model to the distribution of the number of new CLs, we estimated the sample size for clinical trials based on the number of new CLs as the primary outcome and found that randomized clinical trials using this new MRI marker as an outcome are feasible and can be easily pursued.
